# Complete mitochondrial genome of banana skipper *Erionota torus* Evans (Lepidoptera: Hesperiidae) and phylogenetic analysis

**DOI:** 10.1080/23802359.2021.1942264

**Published:** 2021-06-23

**Authors:** Li-Na Liu, Li Zeng, Zhi-Xiang Guo, Bao-Ming Yang, Ke-Suo Yin, Chun-Mei Wang, Si-Jun Zheng

**Affiliations:** a Key Laboratory of Green Prevention and Control of Agricultural Transboundary Pests of Yunnan Province, Agricultural Environment and Resource Research Institute, Yunnan Academy of Agricultural Sciences, Kunming, China; b State Key Laboratory for Conservation and Utilization of Bio-Resources in Yunnan, Yunnan Agricultural University, Kunming, China; c Dehong Tropical Agriculture Research Institute of Yunnan, Ruili, China; d Bioversity International, Kunming, China

**Keywords:** Mitogenome, *Erionota torus*, banana pest, phylogeny

## Abstract

*Erionota torus* (Evans, 1941) is a banana pest and is mainly distributed in Southeast Asia and the Pacific regions. The complete mitogenome of *E. torus* (GenBank accession number MW586888) is 15,987 bp in size, including 13 protein-coding genes, 22 transfer RNAs, 2 ribosomal RNAs genes, and a noncoding A + T-rich region. The A + T-rich region is located between *12S rRNA* and *tRNA^Met^*. The base composition of the whole *E. torus* mitogenome is 39.68% for A, 7.30% for G, 41.55% for T, and 11.47% for C, with a high AT content of 81.23%. The phylogeny analysis indicated that *E. torus* had a close relationship with *Notocrypta curvifascia*. The present data could contribute to the further detailed phylogeographic analysis and provide a comprehensive control strategy for this banana pest.

Banana (*Musa* spp.) is an ideal and low-cost crop and accounts for the fourth most important food in the world today (Tripathi et al. 2021). Especially in developing countries, many populations depend mostly on bananas as food and feed source (Mohapatra et al. 2010). The banana skipper or banana leaf-roller, *Erionota torus* (Evans, 1941) (Lepidoptera: Hesperiidae), is a common banana pest in Southeast Asia and Pacific regions, ranging from Papua New Guinea and Australia, Sikkim to south China, Myanmar, Malaysia, and Vietnam (Inoue and Kawazoe 1970; Okolle et al. 2006; Corbet and Pendlebury 1992). Almost all banana cultivars could be attacked by this skipper (Sivakumar et al. 2014). The larvae of *E. torus* cause considerable damage to banana foliage by rolling the leaf while feeding on it (Chiang and Hwang 1991) and could lead to yield loss of about 20% (Okolle et al. 2010). Elucidating the sequence and structure of *E. torus* mitogenome is important for the diversity and phylogeographic analysis of this banana pest, thus providing information for its comprehensive control.

The specimen of *E. torus* in the present work was obtained from Honghe, Yunnan, China (N 22°77′, E 103°24′), and deposited in the insect museum (handled by Jin-Hua Zhang, email: museum_insect@126.com) in Agricultural Environment and Resources Institute, Yunnan Academy of Agricultural Sciences, with a voucher number AERI-G-20200518. Sequencing work of the complete mitogenome of *E. torus* was performed by Illumina Nextseq500 in Beijing Microread Genetics Co., Ltd., with a total data volume of 4 G (150 bp Reads). High-quality reads were assembled from scratch using IDBA-UD and SPAdes (Gurevich et al. 2013). Protein-coding genes (PCGs) of the *E. torus* mitogenome were identified using BLAST search in NCBI, and tRNA genes were identified using the tRNAscan-SE search server (Schattner et al. 2005). The final assembled mitogenome was also verified on the MITOS web server (Bernt et al. 2013).

The *E. torus* mitogenome is 15,987 bp in size (GenBank accession number MW586888), including 13 typical invertebrate PCGs, 22 transfer RNA genes, 2 ribosomal RNA genes, and a noncoding control region (A + T-rich). The A + T content of the whole *E. torus* mitogenome is 81.23%, showing an obvious AT mutation bias (Eyre-Walker 1997). The A + T-rich region exhibits the highest A + T content (94.89%) in the *E. torus* mitogenome.

All the 13 PCGs use standard ‘ATN’ as start codons and have the common mitochondrial stop codon ‘TAA.’ All the tRNAs except *tRNA^Ser^ (GCU)* could be folded into the typical cloverleaf secondary structures. The unusual *tRNA^Ser^ (GCU)* had completely lost the dihydrouridine (DHU) stem and loop. The 12S rRNA gene is located between *tRNA^Val^* and the A + T-rich region, while the 16S rRNA is located between *tRNA^Val^* and *tRNA^Leul^*. The locations of these two rRNA genes in *E. torus* mitogenome are quite different from the ancestral insect’s mitogenome (Boore 1999).

Based on the concatenated 13 mitochondrial PCGs sequences of 11 species from Hesperiinae, the neighbor-joining method was used to construct the phylogenetic relationship between *E. torus* and 10 other Hesperiinae species (Figure 1). The phylogenetic analysis was performed by MEGA7 software (Kumar et al. 2016). The potential saturation of any PCG was assessed using DAMBE5 software (Xia 2013). The phylogeny analysis indicated that *E. torus* had a close relationship with *Notocrypta curvifascia* (Figure 1), which adds new information to the evolutionary lineage research of *E. torus* (Jaleel et al. 2019). This mitogenome data might be also valuable for further phylogeography analyses and provide a comprehensive control strategy for this banana pest.

**Figure 1.**
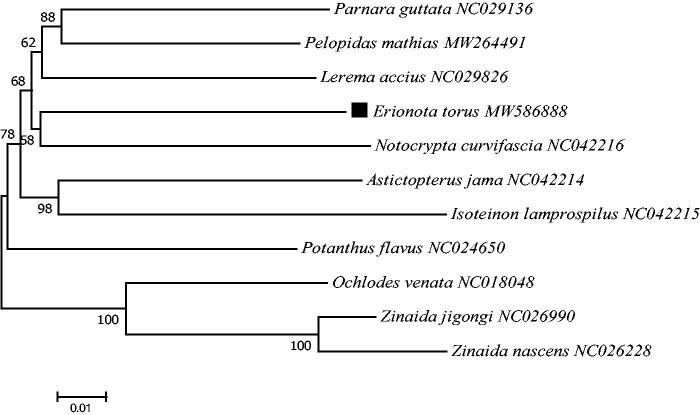
Phylogenetic tree showing the relationship between *E. torus* and 10 other hesperiinae species based on neighbor-joining method performed using 500 bootstrap replicates. *Ochlodes venata*, *Zinaida jigongi* and *Zinaida nascens* were used as outgroups. GenBank accession numbers of each sequence were listed in the tree behind their corresponding species names.

## Data Availability

The genome sequence data that support the findings of this study are openly available in GenBank of NCBI at https://www.ncbi.nlm.nih.gov under the accession no. MW586888. The associated BioProject, SRA, and Bio-Sample numbers are PRJNA721010, SRR14202297, and SAMN18695551 respectively. CRPs are implemented with support from the CGIAR Trust Fund and through bilateral funding agreements. For details, please visit https://ccafs.cgiar.org/donors.
